# Exploring Preeclampsia: A Comprehensive Overview

**DOI:** 10.15190/d.2025.13

**Published:** 2025-09-30

**Authors:** Maheen Nasir, Aimen Binte Asif, Momnah Waheed, Javeria Irfan, Qudsia Umaira Khan, Ayra Waseem

**Affiliations:** ^1^CMH Lahore Medical and Dental College, Pakistan; ^2^Learning Alliance, Pakistan

**Keywords:** preeclampsia, biomarkers, placental growth factor, soluble fms-like tyrosine kinase, placenta.

## Abstract

Preeclampsia remains a significant complication of  pregnancy which emerges after the 20th week mark  and is identified by proteinuria and hypertension.  This review explores the multifaceted nature of  preeclampsia, beginning with its complex pathology  involving endothelial, platelet dysfunction and the  imbalance in the factors that regulate angiogenesis.  Diagnosis relies on monitoring blood pressure and  assessing proteinuria, supported by laboratory tests  and imaging studies to detect organ involvement.  Biomarkers including Soluble fms-like tyrosine  kinase (sFlt-1) and placental growth factor (PlGF)  play a critical role in early detection and risk  stratification. The imbalance in the ratio between  these two biomarkers serves as a key in diagnosing  and predicting preeclampsia. Vascular homeostasis is  upset by this imbalance, which results in clinical  symptoms such as hypertension and urinary protein  excretion. Elevated sFlt-1 and reduced PlGF in high risk pregnancies, including those with chronic  hypertension, correlate with greater clinical severity  and predict adverse outcomes for maternal and fetal  health. Management strategies include the use of  antihypertensive medicines, fetal monitoring and  delivery of the fetus based on disease severity.  Despite ongoing research into predictive biomarkers  and preventative measures, preeclampsia remains a  challenge and necessitates a multidisciplinary  approach for the well-being of both the fetus and the  mother. This review serves as a comprehensive resource for clinicians and healthcare workers and by consolidating current knowledge and practical  approaches allows them to stay updated on the  evolving role of biomarkers in improving diagnostic  accuracy.

## SUMMARY

1. Introduction 

2. Pathogenesis

- 2.1 Placental role in preeclampsia

- 2.2 Placental Hypoxia and Ischemia

- 2.3 Release of Angiogenesis Inhibitors 

- 2.4 Systemic Dysfunction of Endothelium

- 2.5 Immunological and Inflammatory Factors in Preeclampsia

- 2.6 The Impact of the Renin Angiotensin System (RAS) in Preeclampsia

- 2.7 Genetic Contributions to Preeclampsia

3. Diagnosis

3.1 Significance of Biomarkers: sFlt-1/PlGF

4. Complications

- 4.1 Maternal Risks

- 4.2 Fetal Complications

5. Treatment

- 5.1 Antiplanets and Anticoagulant agents

- 5.2 Anti-inflammatory and Immunomodulatory Therapies

- 5.3 Antihypertensives in Preeclampsia

- 5.4 Novel and Targeted Molecular Therapies

- 5.5 Supportive Therapies

6. Conclusion 

## 1. Introduction

Hypertension-related complications of pregnancy affect ten percent of the pregnancies worldwide and are the leading cause of maternal illness and death. These disorders are characterized by new onset hypertension, after the 20th week mark, with a top reading (SBP) ≥ 140 mmHg and a bottom reading (DBP) ≥ 90 mmHg^1^. This includes chronic hypertension which is elevated blood pressure prior to pregnancy or twenty weeks, gestational hypertension which is development of hypertension after twenty weeks in the absence of protein in urine and the more severe pregnancy specific syndromes, preeclampsia and eclampsia. Preeclampsia is a serious disorder that can be life-threatening for pregnant women and typically presents either during or after twenty weeks. This disorder is marked primarily by onset of high blood pressure, proteinuria and signs of end-organ dysfunction in a pregnancy with a previous history of normal blood pressure. Elevated blood pressure must have been recorded separately, on two different occasions, with at least an interval of four hours. In severe preeclampsia, the blood pressure may even be as high as >160mmHg^2^. The urinary protein excretion can be detected and quantified using a 24-hour urine sample or a spot protein to creatinine ratio, where values of 300mg and 0.3, respectively, are required for making a diagnosis^3,4^.

Preeclampsia is grouped into two main types based on when symptoms first appear during pregnancy and the impact on the outcomes for the mother and the fetus. Early onset preeclampsia refers to the beginning of symptoms before 34 weeks and is associated with more severe maternal manifestations. Additionally, poses a greater risk of unfavourable fetal outcomes such as restriction of growth, birth before time, and complications during the neonatal period leading to increased neonatal intensive care unit (NICU) admissions^5^. Contrary to this, late onset occurs after 34 weeks, closer to 37 weeks (term) and presents with milder symptoms as compared to the former. In this type of preeclampsia, the risk of severe fetal complications and maternal harm is low. Therefore, timing of onset may influence the approach to treatment and may alter the timing of delivery, which may be expedited after evaluating the risks of preterm birth against those of continuing with the pregnancy in the presence of preeclampsia^6,7^.

The clinical features and manifestations of preeclampsia can vary widely in severity and presentation because of multi-organ involvement. However, generally they include a combination of the typical signs and symptoms^8^. Women with preeclampsia may present with the complaint of edema in face, hands and feet which is in contrast to the slight swelling common in pregnancy, as it is more marked and sudden in preeclampsia. Visual disturbances such as blurred vision, sensitivity to light (photophobia) or spots and floaters in the visual field are commonly seen due to the systemic vascular abnormalities and endothelial dysfunction, characteristic of preeclampsia. Persistent and recurrent headaches unresponsive to typical remedies and upper abdomen pain due to liver dysfunction are characteristic symptoms of preeclampsia. Serious dysfunction may be indicated by shortness of breath representing pulmonary edema, low urinary output and alterations in mental status and hyperreflexia^9^. All women, however, may not experience all these signs and symptoms and some may significantly overlap with those of a normal pregnancy or other medical conditions^10^.

Preeclampsia is the primary cause of maternal illness and death and ranks among the top causes of maternal demise and poor outcomes. Complicating pregnancies over the world and varying epidemiologically globally, preeclampsia poses a significant concern due to its lethal potential^11^. Regardless of the high prevalence and increased effort into identification of risk factors, there is still a lack of accurate prediction of onset of preeclampsia and the risk is only moderately reduced by preventative therapies^12^. Subsequently, according to the need of better detection and risk assessment, biomarkers have preceded the traditional clinical assessments in the context of preeclampsia and have posed to be valuable tools for early detection and risk assessment. Biomarkers are able to detect the biochemical changes even before clinical symptoms manifest, giving therefore, a more objective and quantitative measurement and sufficient time for early intervention. In addition to being non-invasive or minimally invasive, an additional benefit of biomarkers is the risk stratification; categorising women into groups based on their likelihood to develop preeclampsia. Biomarkers can act as prognostic factors in predicting the severity and progression of preeclampsia and the possibility of severe complications. Commonly studied biomarkers include the markers of endothelial dysfunction such as soluble fms-like tyrosine kinase (sFlt-1) and soluble endoglin, marker of placental insufficiency and promotor of angiogenesis; placental growth factor (PIGF), markers of inflammation such as interleukins^13^. Dysfunction of the endothelium is a result of an imbalance, which is marked by high soluble fms-like tyrosine kinase-1 (sFlt-1) and reduced placental growth factor (PlGF) levels, which is crucial for the development of the placenta and the growth of the vascular endothelial cells. On the other hand, soluble sFlt-1 exacerbates endothelial damage and vasoconstriction by acting as a blocker and antagonist to PIGF and VEGF. Vascular homeostasis is upset by this imbalance, which results in clinical symptoms of preeclampsia such as proteinuria and hypertension. The sFlt-1/PlGF ratio facilitates early diagnosis, which improves diagnostic precision and prognosis evaluation. This helps to guide prompt therapeutic measures to decrease maternal and fetal harm. This ratio is particularly helpful in pregnancies complicated by chronic hypertension, where early identification of superimposed preeclampsia is crucial^42^. Predictive biomarker potential is highlighted by the fact that this dysregulation starts early in pregnancy, with higher sFlt-1 levels detectable weeks before clinical symptoms appear.

In addition to being a diagnostic tool, the ratio not only corresponds to the severity but also informs clinical decisions about when to deliver a patient in order to minimize risks to both the mother and the fetus^42,44,46^.

A comprehensive review on preeclampsia serves an important purpose in increasing our understanding of preeclampsia and the implications it has on maternal and fetal well-being. By synthesizing and amalgamating existing research findings, this review seeks to offer a thorough summary of the latest developments and understanding in the context of preeclampsia. This includes elucidating the pathogenesis of preeclampsia, the diagnostic and management strategies and the recent advancements and potential gaps in the biomarkers for early detection and management of preeclampsia. Such a review is essential for healthcare workers to enhance early detection, improve pregnancy outcomes and aid in effective management. It further adds to earlier reviews by comparing findings across studies and emphasizing the current limitations to application of biomarkers in routine practice.

## 2. Pathogenesis

**Figure 1:**
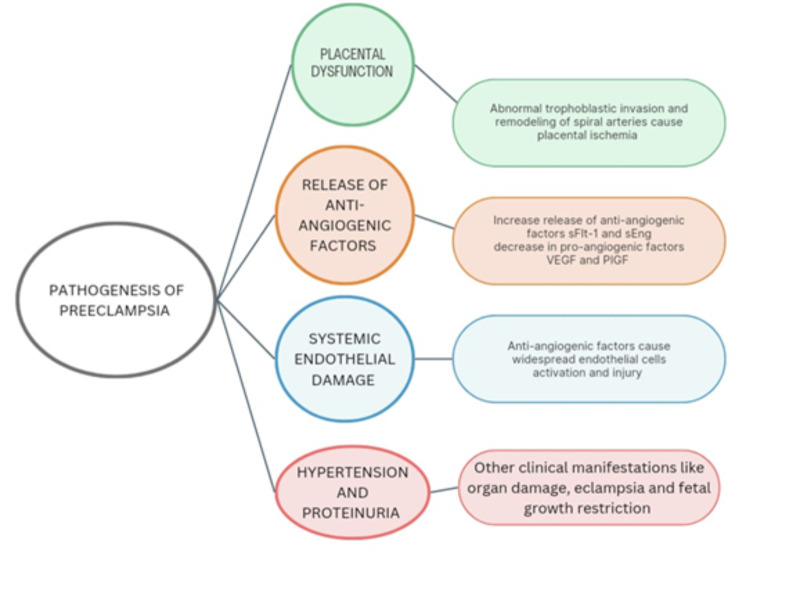
Pathogenesis of Preeclampsia 15, 16, 17,18, 19

The biological progression of preeclampsia involves multifaceted processes which ultimately culminate in complications like increased blood pressure and proteinuria in pregnant females (Figure 1).

Preeclampsia is a prevalent pregnancy condition that begins in the placenta and is marked by poor invasion of cytotrophoblastic cells and extensive dysfunction of maternal blood vessels^14^. This condition results in the overproduction of vascular growth inhibiting factors from the placenta into the maternal bloodstream, resulting in hypertension, proteinuria, and a range of other symptoms. Although the specific molecular mechanisms are not fully understood, researchers suggest that hypoxia, inflammation and oxidative damage all contribute to the pathogenesis^15^.

2.1. Placental Role in Preeclampsia

The placenta is the central factor in the etiology of preeclampsia, as resolution of symptoms depends on its removal^16^. Preeclampsia is linked to abnormal development of placental vasculature. In standard placental maturation, cytotrophoblast engage in pseudo-vasculogenesis, to emulate an endothelial phenotype. The cells from placenta invade the maternal blood vessels in the uterus, converting them into larger vessels to guarantee sufficient placental perfusion. This transformation entails the suppression of cell adhesive molecules and the assumption of characteristics like vascular lining cells. However, in preeclampsia, this invasion is insufficient because cytotrophoblastic cells fail to undergo such modification resulting in high-resistance vessels and inadequate placental perfusion^17^.

2.2. Placental Hypoxia and Ischemia

 Due to the inadequate blood supply, the placenta becomes hypoxic (low oxygen). Hypoxia triggers the secretion of various agents that contribute to systemic inflammatory response and endothelial dysfunction^18^. Oxidative stress is also increased by hypoxia which leads to vascular injury and further amplification of maternal inflammatory response.

2.3. Release of Angiogenesis

Inhibitors Vascular growth mediators and their receptors are crucial in placental vascular development. Disrupted signaling within these pathways is observed in preeclampsia. Elevated levels of vascular growth inhibitor like sFlt-1 disrupt normal angiogenesis, leading to shallow cytotrophoblast invasion and placental ischemia. Additionally, TGF-β, which inhibits cytotrophoblast invasion, is overexpressed in preeclampsia^19^. These anti-angiogenic factors enter into the maternal circulation and contribute to hypertension and multi-organ dysfunction. The imbalance between pro-angiogenic and anti-angiogenic mediators is considered the be the hallmark mechanism which drives the disease progression.

2.4. Systemic Endothelial Dysfunction

The imbalance of vascular growth mediators, notably heightened expression of sFlt-1 alongside decreased levels of PlGF and VEGF plays a pivotal role in endothelial dysfunction^20^. Originating from the placenta, sFlt-1, a truncated VEGF receptor, disrupts normal vascular function by sequestering VEGF and PlGF^21^. Elevated sFlt-1 levels often precede preeclampsia and correlate with its severity. VEGF, crucial for maintaining endothelial stability becomes deficient particularly in vital organs inducing organ impairment^22^. Furthermore, the reduction of PlGF, which enhances VEGF-mediated signaling, plays a part in the progression of preeclampsia. Additionally, the presence of soluble endoglin (sEng), another antiangiogenic factor, exacerbates vascular injury and is elevated in preeclampsia, often manifesting before clinical symptoms emerge^23^. Nitric oxide (NO), an essential vasodilator, is also impaired in preeclampsia, with diminished production associated with elevated levels of sFlt-1 and sEng. This intricate interplay of factors ultimately culminates in the characteristic endothelial dysfunction and subsequent organ damage observed in preeclampsia^24^.

2.5. Immunological and Pro-Inflammatory Agents in Preeclampsia

Inflammation and immune dysregulation make a significant contribution to obstructing the penetration of trophoblast into uterine lining. This condition exhibits a greater prevalence among women with no previous pregnancies, those with altered parental status, and those with prolonged interpregnancy intervals, indicating the involvement of immune related mechanisms^25^. Notably, untreated HIV positive women demonstrate a reduced occurrence of preeclampsia, which escalates with the initiation of antiretroviral therapy^26^. Preeclampsia is marked by upregulation of placental macrophages and chemokines, fostering an inflammatory

**Table 1:** Diagnostic Criteria based 2003, 2013 and 2018 ACOG guidelines ^12^,36

CRITERION	2002 ACOG GUIDELINES	2013 ACOG GUIDELINES	2018 ACOG GUIDELINES
Hypertension Definition	SBP is equal to or higher than 140mmHg and DBP is equal to or higher than 90mmHg on two separate occasions, taken 6 hours apart	SBP is equal to or higher than 140mmHg and DBP is equal to or higher than 90mmHg on two separate occasions, taken 4 hours apart	SBP is equal to or higher than 140mmHg and DBP is equal to or higher than 90mmHg on two separate occasions, taken 4 hours apart
Proteinuria Definition	≥ 300 mg/24 hours or ≥ 1+ dipstick	≥ 300 mg in urine sample over 24 hours, protein/creatinine equal to or higher than 0.3 or ≥ 1+ dipstick	≥ 300 mg in urine sample over 24 hours, protein/creatinine equal to or higher than 0.3 or ≥ 1+ dipstick
Alternative Diagnostic Criteria	Not mentioned	In absence of proteinuria and signs of end-organ dysfunction	In absence of proteinuria and signs of end-organ dysfunction
Timing of Diagnosis	After 20 weeks	After 20 weeks	After 20 weeks
Emphasis on Role of Biomarkers	Not specified/included	sFlt-1 and PIGF mentioned but not standardized	Recommended to rule out sFlt-1 and PIGF before 37 weeks
Recommendat ions for Management	Deliver at 37 weeks in mild cases or earlier in severe cases	Deliver at 37 weeks in mild cases, analysis in individual, severe cases	Deliver at 37 weeks in mild cases, analysis in individual, severe cases .
Postpartum Monitoring	Monitor blood pressure and observe symptoms	Continue antihypertensive medications, monitor for 72 hours postpartum and follow up	Continue antihypertensive medications, monitor for 72 hours postpartum and follow-up

microenvironment that impairs trophoblast invasion and the remodeling of spiral arteries. Moreover, syncytiotrophoblast debris facilitates maternal vascular impairment and the heightened levels of sFlt-1 and sEng^27^. Genetic predispositions, such as the KIR-AA genotype in maternal natural killer (NK) cells and the fetal HLA-C2 gene variant in fetus, may also augment the vulnerability to preeclampsia^28^. Additionally, oxidative stress within the placenta, marked by increased lipid peroxidation and the generation of free radicals, is associated with preeclampsia, although antioxidant supplementation has not yielded significant risk reduction^29^.

2.6. The Impact of the Renin Angiotensin System (RAS) in Preeclampsia

In preeclampsia, the renin-angiotensin system (RAS) exhibits suppression. Typically, aldosterone and angiotensin levels elevate during pregnancy; however, in women suffering from preeclampsia, plasma levels of renin are diminished, accompanied by heightened sensitivity to angiotensin II and other vasoconstrictors^30^. Angiotensin II exerts its hypertensive effects through arterial vasoconstriction via receptor binding. In preeclampsia, this increased sensitivity may arise from autoantibodies that activate the angiotensin II receptor, thereby reducing trophoblast invasiveness and precipitating damage to placenta and heightened levels of sFlt-1^31^.

2.7. Genetic Contributions to Preeclampsia

While most cases of preeclampsia occur without a hereditary background, having an immediate relative with this condition increases the likelihood of severe preeclampsia by two to four times. Additionally, if a woman conceives with a man who has a prior history of fathering a pregnancy complicated by preeclampsia, her risk nearly doubles^32,33^. These findings suggest a significant paternal (and fetal) genetic component, potentially following a monogenic inheritance pattern requiring homozygous condition for a recessive allele shared by both maternal parent and fetus^20^. Alternatively, genomic imprinting might play a role. STOX1, a gene extensively studied in this context, has yielded inconsistent associations with preeclampsia. Moreover, preeclampsia is likely polygenic, influenced by multiple susceptibility genes such as prothrombin, angiotensin converting enzyme, endoglin and apolipoprotein A1^34^.

## 3. Diagnosis

The World Health Organization (WHO) guidelines for preeclampsia date back to 1987 and diagnostic criteria for preeclampsia was defined as proteinuria in addition to the presence of gestational hypertension. The former was characterized as excretion of 0.3 g of protein in either mid-stream or a twenty-four hour urinary sample. The 2002 guidelines by The American College of Obstetricians and Gynecologists (ACOG) closely resemble those presented by WHO, with organ dysfunction still being recognized as the feature of advanced form of preeclampsia.^35^Almost a decade later in 2013, in updated guidelines, ACOG removed the emphasis on proteinuria for diagnosing preeclampsia and considered end-organ derangement and dysfunction significant enough. This emphasis was again reiterated in the 2018 (ACOG) guidelines which remained largely unchanged from the guidelines published in 2013^12,36^.

Diagnostic Criteria based on 2003, 2013 and 2018 ACOG guidelines includes elevation of blood pressure, urinary excretion of protein and signs of end-organ dysfunction such as a marked fall in the level of platelets, impaired function of the liver and kidney, buildup of fluid in lungs and recent onset of visual and cerebral disturbances. The guidelines specified specific thresholds for aiding in the detection of preeclampsia (Table 1).

Diagnostic approaches have been furthered refined by recent guidelines. The NICE 2019 guideline emphasizes diagnosis of preeclampsia when hypertension occurs after 20 weeks of gestation and is accompanied with significant proteinuria (>300mg/24hrs). The guideline emphasizes the detection of uteroplacental dysfunction using routine blood pressure measurements and assessment of urinary protein alongside fetal growth and Doppler studies. The ISSHP 2021 statement broadened the diagnostic framework and no longer required proteinuria for diagnosis and instead emphasized on diagnosing preeclampsia when hypertension was accompanied by maternal organ dysfunction, acknowledging that maternal and fetal morbidity can occur even in the absence of proteinuria^13,37^.

Considering the fact that hypertension is a hallmark of preeclampsia, measurement of the blood pressure is essential for detection. It is considered to be elevated if, if SBP is equal to or higher than 140 and DBP is equal to or higher than 90. With emphasis on the fact that the reading must have been repeated twice, on different occasions, at least with an interval of four hours^38^. Proper examination and evaluation of the patient for the common symptoms of preeclampsia which include persistent headaches, nausea, vomiting, vision changes (such as blurring or eye floaters), pain in the upper part of the abdomen and rapid increase in weight or swelling, signifying edema, particularly in the face and hands. According to current guidelines, in the early pregnancy, maternal history and characteristics can be used to stratify women into a group with high risk, but this method has a relatively low sensitivity^39^. Although not yet implemented into routine practice because of financial barriers, a better and more reliable screening method for first trimester detection would include mean arterial blood pressure measurements, Doppler USG and measurement of the proangiogenic factor, placental growth factor (PIGF) in the blood^40^. Detection of proteinuria is a key component in the diagnosis of preeclampsia where significant proteinuria is indicated if excretion is of 300mg or more in a 24 hour sample, a urine protein to creatinine ratio (uPCR) ≥ 0.3 or more or a dipstick reading of 1+ or greater, though the latter is less reliable than the other methods of quantification^3,41^. Blood tests including a complete blood count, liver function tests and renal function tests, play a vital role in detecting complications such as hemolysis, thrombocytopenia, liver enzyme abnormalities, and derangement of renal function, which is signified by elevated creatinine and impaired glomerular filtration rate (GFR). Imaging studies, particularly ultrasound (USG), are crucial as assessment of fetal growth, measurement of amniotic fluid volume and umbilical artery Doppler can be used to detect oligohydramnios and restricted fetal growth.

3.1. Significance of Biomarkers: sFlt-1/PlGF

Reduced levels of PIGF in the maternal circulation and increased levels of sFlt-1 produced from the placenta are indicative of an angiogenic imbalance, which is one of the pathophysiology of preeclampsia. When combined with other clinical indicators including high blood pressure and proteinuria, these biomarkers have shown effective in the diagnosis, prognosis, and clinical decision-making process^42^.

Nevertheless, the majority of prognostic models constructed with sFlt-1, PIGF, or their ratio rely on threshold values to dichotomize the continuous observations of these markers^43^ (Table 2). In addition to being useful in the diagnosis of individuals presenting with unusual manifestations of preeclampsia, we suggest that these two biomarkers and the sFlt-1/PIGF ratio may also be useful in predicting preeclampsia in chronically hypertensive women at risk of developing superimposed preeclampsia.^44^One crucial component in the therapeutic treatment of preeclampsia is placental induced growth factor (PIGF). PIGF, a member of the vascular endothelial growth factor family, is primarily released by the placenta and is also to some degree by other tissues such as heart, bone, thyroid, liver, lungs. Vascular Endothelial Growth Factor (VEGF) activity is regulated by PIGF, which binds competitively to its receptor (VEGFR-1), which permits VEGF to bind, and then to VEGFR-2, that has potent tyrosine kinase activity. The placenta secretes PIGF primarily during pregnancy, aiding in the growth and strengthening of the placental vasculature. Placental development and PIGF secretion rise starting from the second trimester of pregnancy^45^. During early stages, antiangiogenic factors like sEng and sFLT-1 play crucial roles in endothelial function and vascular remodeling. These factors, particularly sFLT-1, inhibit the action of PIGF and VEGF, leading to dysfunction of the endothelium. In normal pregnancies, levels of sFLT 1 decrease and PIGF increases by the second trimester, promoting angiogenesis. However, in preeclampsia, there is an imbalance with elevated sFLT-1 and sEng levels and decreased PIGF and VEGF levels. This imbalance disrupts maternal endothelial homeostasis, contributing to hypertension, proteinuria, and other symptoms of preeclampsia. Research suggests that higher levels of sFLT-1 are associated with the severity of preeclampsia and can serve as a reliable predictor^46^. In singleton pregnancies, the sFlt-1/PLGF level corresponds with the meantime till delivery (MTUD); a highly raised ratio may suggest that delivery is necessary within 48 hours^47^. PlGF levels are downregulated and circulating maternal blood levels of sFlt-1 are increased in preeclampsia. sFlt-1, a blocker of the pro-angiogenic factors, induces damage to the endothelium and vasoconstriction, which can result in preeclampsia and fetal development limitation. This change is present before the illness manifests and persists during the illness. In preeclampsia, sFlt-1 increases around 5 weeks before the illness manifests, while PlGF levels start to fall before sFlt-1 does. Therefore, a number of studies indicate that this ratio is a more accurate predictor of preeclampsia diagnosis as compared to sFlt-1 or PlGF measurement alone. Estimation of the ratio is associated with unfavorable pregnancy and postpartum outcomes in addition to predicting the start of preeclampsia^48^(Table 2). Although the use of biomarkers has shown clear benefits in improving diagnostic accuracy and thus facilitating earlier intervention in preeclampsia, the widespread clinical

**Table 2:** Risk Stratification of Preeclampsia based on sFlt-1: PlGF ratio ^44-47^

Range	Risk Category	Clinical Implications
< 38	Low risk	The likelihood of developing preeclampsia within the next week is low. Monthly assessment after 20 weeks if clinically appropriate. 2% risk of developing preeclampsia (PROGNOSIS trial).
38–85 (early onset preeclampsia)	Intermediate risk	Enhanced monitoring recommended. Repeat the test after 1–2 weeks or sooner if the clinical situation worsens. Provides additional risk assessment for preeclampsia development within 4 weeks.
38–110 (late onset preeclampsia)	Intermediate risk	Enhanced monitoring recommended. Repeat the test after 1–2 weeks or sooner if the clinical situation worsens. Provides additional risk assessment for preeclampsia development within 4 weeks.
> 85 (early-onset preeclampsia)	High risk	Intensive monitoring required. Immediate attention and management to mitigate preeclampsia risks. Highest likelihood of developing or already having preeclampsia.
>110 (late-onset preeclampsia)	High risk	Intensive monitoring required. Immediate attention and management to mitigate preeclampsia risks. Highest likelihood of developing or already having preeclampsia

application is limited by heterogeneity across studies. The implementation is hindered by high costs, restricted accessibility and limitation in validation across various populations. Across studies, a variability in the diagnostic threshold is observed which highlights the need for further validation and analysis before routine adoption.

## 4. Complications

4.1. Maternal Risks

Preeclampsia presents immediate and long-term dangers to maternal well-being:

Eclampsia: Seizures can arise in preeclamptic women, leading to eclampsia, a severe condition if not promptly addressed^49^.

Hemolysis, Elevated Liver enzymes and Low Platelets (HELLP) Syndrome: This condition could potentially leading to liver failure, bleeding issues, and other critical complications^50^.

Renal Impairment and Liver Damage: Preeclampsia can result in renal impairment and liver damage, affecting maternal health and requiring careful management^51^.

Placental detachment and pulmonary edema: Detachment of the placenta and pulmonary edema are potential complications of preeclampsia, necessitating prompt medical attention.

Increased Risk of Maternal Mortality:Eclampsia poses a risk of maternal mortality, particularly among older pregnant women, with reported rates ranging from 10-15%.

Long-term Health Hazards: Preeclampsia heightens the risk of cardiovascular diseases later in life. It also elevates likelihood of kidney disease, diabetes, and metabolic syndrome. Women who have experienced preeclampsia in past face increased odds of recurrence in subsequent pregnancies^52^.

4.2. Fetal Complications

Preeclampsia can lead to significant problems:

Intrauterine Growth Restriction (IUGR): Diminished blood flow to the placenta due to preeclampsia may result in IUGR, leading to decreased birth weight and premature birth^49^.

Preterm Birth: Preeclampsia often necessitates premature delivery, heightening the risk of complications such as respiratory distress syndrome, intraventricular hemorrhage, and NICU admission^53^.

Fetal distress and emergency delivery: Severe preeclampsia may trigger fetal distress requiring emergency delivery, posing risks to both mother and baby.

## 5. Treatment

Preeclampsia has a variety of different treatment programs that aim to improve both maternal and fetal outcomes as summarized in (Table 3).

**Table 3:** Treatment Modalities for Preeclampsia based on underlying pathology

Pathology	Therapeutic strategy
Pathology	Therapeutic strategy
Vasoconstriction	Aspirin, Sildenafil, Nifedipine, Hydralazine, Sulfasalazine, Methyldopa, Labetalol
Inflammatory response	Aspirin, Celecoxib, Hydroxychloroquine, Statins, Eculizumab, Enteracept, Nifedipine, MSC transplant
Hypercoagulability	Aspirin, LMWH, IV Antithrombin III
sFlt1 and sENG production	Statins, Metformin, Enteracept, Hydralazine, Sulfasalazine, Apheresis, siRNA, VEGF A/VEGF-B
Reactive oxygen species production	Metformin, PPIs, Vitamins, Polyphenols, Probiotics
Decreased uteroplacental blood flow	Epidural therapy

**Figure 2:**
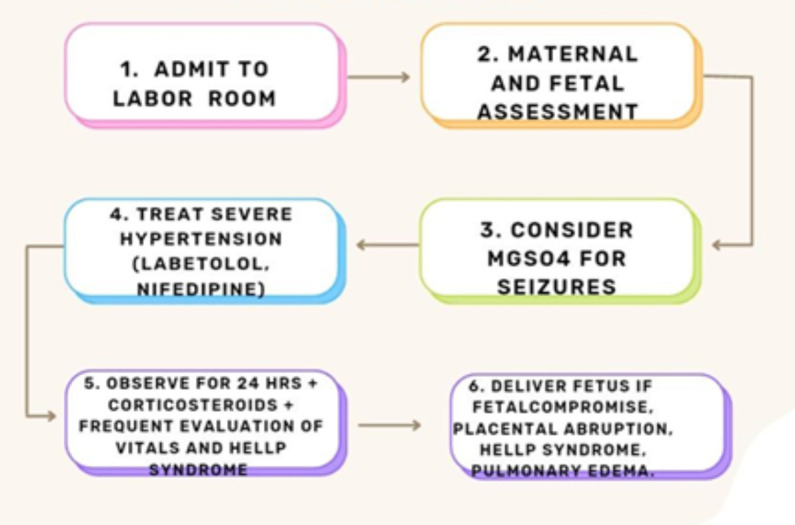
Management of Preeclampsia

Major efforts are in progress to understand the mechanisms and pathophysiology of preeclampsia and so the treatment is still undefined. The last resort, however, to manage preeclampsia continues to be delivery of the infant (Figure 2).

Several pharmacological therapies have been considered for their potential role in modifying the course of preeclampsia. These therapies target the different pathogenic pathways of preeclampsia including platelet aggregation, endothelial dysfunction, angiogenic imbalance and the exaggerated inflammatory response. Varying degrees of clinical evidence support the established agents such as aspirin, heparin and the conventional anti hypertensive agents. Whereas, other modalities such as metabolic agents, targeted molecular therapies and immunomodulators are currently largely experimental.

5.1. Antiplatelet and Anticoagulant Agents

By targeting platelet activation and the coagulation cascade, antiplatelets and anticoagulant agents improve placental perfusion and reduce the risk of maternal complications. Aspirin remains the most widely accepted prophylactic therapy, whereas heparin has been proposed as an adjunct (Table 4).

**Table 4:** Antiplatelet and anticoagulant therapies^54^,^55^

Class/Drug	Proposed Mechanism/ Effect
Aspirin	Low dose (100-160mg) restores the PGI2/TXA2 balance and reduces vasoconstricting prostaglandins. Most effective if initiated < 16 weeks gestation^54^
Heparin (LMWH)	Inhibits complement’s effect on the trophoblast and positively effects the maternal vasculature^55^

5.2. Anti-inflammatory and Immunomodulatory Therapies

In preeclampsia, a key role is played by inflammatory dysregulation. Several anti-inflammatory agents including conventional immunomodulators and newer biologics, have been investigated for their ability to reduce inflammatory cascade, to improve endothelial integrity and to modulate angiogenic factors (Table 5).

**Table 5:** Anti-inflammatory and immunomodulatory therapies in pre-eclampsia ^56-62^

Class/Drug	Proposed Mechanism/Effect
Hydroxychloroquine	An antimalarial which reduces the incidence of preeclampsia in SLE pregnancies
Statins	Inhibit the activity of HMG CoA reductase Increase hepatic LDL receptors
	Block secretion of pro inflammatory cytokines Reduce the inflammatory cascade by lowering LDL, which activates Toll-like receptors that increase IL-6 and TNF-a Modulate angiogenic factos by increasing release of sFlt-1 and sEng from trophoblastic cells, which bind VEGF, PIGF, TGF b and thus inhibit their action Improves vasodilation and lower blood pressure by increasing eNOS on endothelial cells
Sulfasalazine	Inhibit NFkB activity and cytokines; upregulate eNOS and lower SFlt-1 (under investigation)
Celecoxib	COX-2 inhibitor; reduces PGE2 and gestational hypertension
Proton pump inhibitors	Antioxidant; upregulate haem oxygenase 1; reduce placental ischemia-induced hypertension
Etanercept	TNF-a inhibitor; lowers sFlt-1 and ROS

5.3. Antihypertensives in Preeclampsia

The cornerstone of symptomatic management in preeclampsia is blood pressure control. Standard anti hypertensives work beyond their hemodynamic actions and exert additional affects on angiogenic and inflammatory pathways (Table 6).

**Table 6:** Antihypertensives in preeclampsia ^63-65^

Class/ Drug	Proposed Mechanism/ Effect
Methyldopa	Alpha-2 receptor agonist; reduces blood pressure by inhibiting sympathetic outflow
Hydralazine	Direct vasodilator; reduces sFlt-1, IL-6 and TNF-a
Nifedipine	Calcium channel blocker; NFkB modulation which reduces IL-1, IL-6, COX-2 and TNF-a
Labetalol	Beta blocker; increases bioavailability of endothelial NO

5.4. Novel and Targeted Molecular Therapies

Latest developments focus on correcting the angiogenic imbalance and complement activation. Currently largely experimental, these targeted therapies highlight the potential for disease modifying interventions (Table 7).

**Table 7:** Targeted therapies under investigation for pre-eclampsia ^59^,^66-70^

Class/ Drug	Proposed Mechanism/ Effect
Eculizumab	Monoclonal antibody to complement component C5, inhibits complement activation
sFlt-1 Aphersis	Removes circulating sFlt-1; lowers proteinuria and maternal blood pressure
siRNA therapy	selectively inhibit specific proteins; siRNA for sFlt1 protein reduce its levels by >50%
VEGF-A/B Peptides	Bind sFlt-1; restore VEGF signalling; improve maternal blood pressure
Sildenafil	PDE-2 inhibitor; increases NO-cGMP pathway; improves placental perfusion
Antithrombin III	Corrects antithrombin deficiency; reduces hypercoagulability and prolongs pregnancy

5.5. Supportive Therapies

Several supportive approaches have been proposed which aim to improve maternal endothelial integrity and fetal growth (Table 8).

**Table 8:** Supportive and emerging therapeutic options in pre-eclampsia ^71-74^

Class/Drug	Proposed Mechanism/Effect
Oral probiotics	Correct vaginal dysbiosis; the deficiency of Lactobacillus crispatus causes disruptions during pregnancy
Antioxidants	Vitamins/polyphenols reduce placental ROS; improve endothelial function
Mesenchymal Stem Cells	Immunosuppressive effect on T lymphocytes; reduced inflammatory cytokines, proteinuria and blood pressure
Epidural Therapy	Prolonged therapy (> 1 week) improves placental blood flow and reduces DBP; reduces the risk of kidney and liver failure;

## 6. Conclusion

In conclusion, this article on preeclampsia provides a comprehensive overview of this intricate pregnancy related condition, highlighting its profound effects on both the health of mother and baby. The review elucidates multifactorial origins of preeclampsia, incorporating genetic, immunological, and environmental influences. It details the current diagnostic criteria, which include monitoring blood pressure and assessing proteinuria, and also explores emerging biomarkers that hold promise for early detection. The discussion also emphasizes the importance of timely intervention and management strategies to mitigate risks such as antihypertensive therapy and delivery planning. By providing a thorough understanding of preeclampsia, the article aims to enhance awareness and guide healthcare professionals in improving outcomes for affected women and their babies.
